# Letter on Homœopathy

**Published:** 1859-10

**Authors:** 


To the Editor of the Buffalo Medical Journal.

*My Dear Sir*,—" A remarkable obstetrical case " in your last Journal reminds me of another " case," which may at least be plead in palliation of your temerity, in charging the "patronage of medical humbug" on "the most educated people." I had for many years been the family physician of a gentleman of wealth and intelligence, classically and thoroughly educated, and now occupying one of the highest and most responsible official positions in the State; and, according to their own assurance, I had the entire confidence of the family. Nevertheless, I was finally supplanted by homœopathy. I was even then urged to waive my repugnance to homœopathy in their favor, so far as to officiate as their *diagnosticator*, that they might be enabled to select their own pellets appropriately. Of course, I declined the honor.

A few years had intervened when I was called in haste, and found the family in great alarm, evidently regarding the wife, a most estimable lady, as in a very dangerous, if not dying condition. She was laboring for breath, with a purple and turgid countenance, a depressed pulse, and really seemed in the agony of suffocation. She had pneumonia, occupying both lungs, and had been several days under homœopathic treatment. The case was urged upon me, with the assurance that I should have the entire control of it. This was at 8 o'clock, A. M. I spoke favorably of the prospect of success under appropriate treatment, for her encouragement, though I regarded the case as doubtful. She was bled moderately from the arm, scarified and cupped freely, blistered, put under the influence of calomel and emetic tartar in combination with opium; and at 4 o'clock, P. M. she was comparatively comfortable; and I felt justified in assuring her that both the urgency of disease, and the terrors of allopathy were past. Having several patients in the vicinity, I was forced to neglect them for the *eight hours* I had been in constant attendance on her, and I left her with the assurance that I would be absent only a short time. I was gone—thirty to forty minutes—and on my return found homœopathy reinstated. "She had lost her 'confidence' in '*allopathy*,' and returned to her first love." There was no change in the symptoms of the disease, but valiant

"confidence" had reversed its usual order of march, and had taken to its heels on the first disappearance of danger. The subsequent treatment is unknown to me; but my successor was not ignorant of mine, while she was in my hands. The probable solution of the matter is that pride was covertly at work, and shrank from the ignominy which might follow the known discomfiture of its petted humbug; and therefore this adroit movement was adopted to cover the retreat between the two horns of the dilemma, death and disgrace. The recovery went on, and the husband, sometime afterwards, gave me the very complimentary assurance, that "he was not quite certain that my treatment of his wife's case might not have had *some* influence in the happy result." There has been much occasion since in the family for homœopathy, but no deaths—probably no apprehesion of such a result. It remains to be seen whether "confidence" in homœopathy will stand fire next time.

A year perhaps after these occurrences, I was called to attend the lady in labor, with the notice that I was wanted *merely* as *obstetrician.* I went, and found a most embarrassing case of shoulder presentation, which resulted safely, both to mother and child. Did I dishonor my profession by answering the call? L.

April, 1859.

The above communication has been upon our table for some months, but was laid away with other papers when we removed to New York. This is an account of one of the numerous instances of insult to which our profession is subjected, we are charitable enough to believe, thoughtlessly, by those from whom we have a right to expect a different course. The now well-established fact that many diseases, formerly regarded as quite serious, are self-limited; and that some of the most serious, frequently have got well and do get well in spite of injudicious treatment even, offers a satisfactory explanation to the physician, of the so-called success of homœopathists in practice. This cannot be made so evident, however, to others, and it is useless to argue the point with them. The question with which our correspondent concludes, seems to us to be a matter to be decided entirely by his own feeling. The honor of our profession would certainly be involved were we ever to meet irregular practitioners professionally; but it is often impossible to absolutely refuse to visit a patient and must always be painful, especially when we are asked to go in the name of common humanity. This is particularly so in cases of labor, a condition in which a woman naturally excites all our sympathy. For a physician to refuse the aid of his skill in a case of difficult labor, under any circumstances, must be exceedingly distressing; nevertheless, in instances like that mentioned by our correspondent, we think that a physician would be justified in an unconditional refusal. The heart must sometimes overrule the head.

